# Dissecting the shared genetic architecture between Alzheimer’s disease and frailty: a cross-trait meta-analyses of genome-wide association studies

**DOI:** 10.3389/fgene.2024.1376050

**Published:** 2024-04-19

**Authors:** Nitesh Enduru, Brisa S. Fernandes, Zhongming Zhao

**Affiliations:** ^1^ Center for Precision Health, McWilliams School of Biomedical Informatics, The University of Texas Health Science Center at Houston, Houston, TX, United States; ^2^ Department of Epidemiology, Human Genetics and Environmental Sciences, School of Public Health, The University of Texas Health Science Center at Houston, Houston, TX, United States; ^3^ Faillace Department of Psychiatry and Behavioral Sciences, McGovern Medical School, The University of Texas Health Science Center at Houston, Houston, TX, United States

**Keywords:** frailty index, frailty phenotype, GWAS, colocalization, Mendelian randomization, genetic correlation, gene association, pathway analysis

## Abstract

**Introduction:** Frailty is the most common medical condition affecting the aging population, and its prevalence increases in the population aged 65 or more. Frailty is commonly diagnosed using the frailty index (FI) or frailty phenotype (FP) assessments. Observational studies have indicated the association of frailty with Alzheimer’s disease (AD). However, the shared genetic and biological mechanism of these comorbidity has not been studied.

**Methods:** To assess the genetic relationship between AD and frailty, we examined it at single nucleotide polymorphism (SNP), gene, and pathway levels.

**Results:** Overall, 16 genome-wide significant loci (15 unique loci) (*p*_meta-analysis_ < 5 × 10^−8^) and 22 genes (21 unique genes) were identified between AD and frailty using cross-trait meta-analysis. The 8 shared loci implicated 11 genes: *CLRN1-AS1*, *CRHR1*, *FERMT2*, *GRK4*, *LINC01929*, *LRFN2*, *MADD*, *RP11-368P15.1*, *RP11-166N6.2*, *RNA5SP459*, and *ZNF652 *between AD and FI, and 8 shared loci between AD and FFS implicated 11 genes: *AFF3*, *C1QTNF4*, *CLEC16A*, *FAM180B*, *FBXL19*, *GRK4*, *LINC01104*, *MAD1L1*, *RGS12*, *ZDHHC5*, and *ZNF521*. The loci 4p16.3 (*GRK4*) was identified in both meta-analyses. The colocalization analysis supported the results of our meta-analysis in these loci. The gene-based analysis revealed 80 genes between AD and frailty, and 4 genes were initially identified in our meta-analyses: *C1QTNF4*, *CRHR1*, *MAD1L1*, and *RGS12*. The pathway analysis showed enrichment for lipoprotein particle plasma, amyloid fibril formation, protein kinase regulator, and tau protein binding.

**Conclusion:** Overall, our results provide new insights into the genetics of AD and frailty, suggesting the existence of non-causal shared genetic mechanisms between these conditions.

## Introduction

Alzheimer’s disease (AD) and frailty are two profound health concerns that exert a substantial impact on the aging population. AD is characterized by a relentless neurodegenerative process resulting in cognitive deterioration, memory loss, and alterations in behavior and physical capabilities. It affects approximately 6.7 million Americans aged 65 and above (Alzheimers Dement, 2023). Concurrently, frailty represents a clinically identifiable state of increased vulnerability and decreased physiological reserves in older adults in everyday life (Chen et al., 2014). There are several frailty definitions; the most common ones are the frailty phenotype (FP) (Fried et al., 2001) and the frailty index (FI) (Mitnitski et al., 2001). FP is diagnosed as a clinical syndrome predicated upon the presence of three of five physical components, namely, weakness, slow walking speed, inadequate physical activity, exhaustion, and unexpected weight loss. On the contrary, FI is rooted in accumulating various health deficits throughout an individual’s lifetime.

The pathophysiological underpinnings of AD are characterized by intricate and multifactorial processes involving beta-amyloid plaques and neurofibrillary tangles. Studies have illuminated the pivotal roles of inflammation and oxidative stress in the initiation and progression of AD and frailty (Di Bona et al., 2010; Chen et al., 2014; Sargent et al., 2018). Furthermore, these conditions share common risk factors such as age, cognitive decline, slow walking, depression, and diabetes. Notably, frailty is a recognized risk factor for AD (Buchman et al., 2007; Ward et al., 2022). With improvements in lifestyle and advanced therapeutics, the extended life span of the population has amplified the prevalence of both AD and frailty. AD prevalence increases with age, being 5.0% from age 65 to 74, 13.1% from age 75 to 84, and 33.3% from age 85 and older, respectively (Rajan et al., 2021). Estimating the prevalence of FI is challenging, but it is estimated to be 18% worldwide in the population aged 60 or above (Siriwardhana et al., 2018). Consequently, many studies used the FP, which is easier to ascertain in large population studies, to assess the prevalence of frailty. In the United States, for the population older than 65 years, the frailty prevalence ranges from 6% to 12% and increases from 3.9% in the 65–74 years age group to 25% in those older than 85 years (Fried et al., 2004). The comorbidity of AD and frailty can further impair quality of life (Mhaolain et al., 2012) and amplify healthcare costs (Butler et al., 2016).

A systematic review and meta-analysis of frailty in mild to moderate AD suggested an increased prevalence of frailty in AD (Kojima et al., 2017), and a randomized clinical trial showed a greater prevalence of frailty in AD compared to mild cognitive impairment (MCI) (Wightman et al., 2023). Although there are no genetic studies directly related to AD and frailty, some epidemiological studies have found an association between AD and frailty (Gomez-Gomez and Zapico, 2019; Alvarado et al., 2021; Sabbatinelli et al., 2021), and AD biomarkers and frailty (Koch et al., 2013; Wallace et al., 2018; Canevelli et al., 2021). Genome-wide association studies (GWAS) for AD and frailty have identified single nucleotide polymorphism (SNPs), genes, and susceptibility loci. Findings from these GWAS have suggested shared genetic underpinnings between the risk factors common to both AD and frailty, such as body mass index (BMI), cardiovascular diseases, depression, and smoking. To the best of our knowledge, no genetic studies have investigated potential pleiotropy or shared underlying mechanisms between AD and frailty using either SNP or gene-level information from GWAS of these two traits.

In the current study, we carried out comprehensive analyses with the goal of exploring the genetic and potential causal relationship between AD and frailty. Our investigation revealed a nuanced genetic overlap with minimal genetic correlation between AD and frailty. Furthermore, we employed cross-trait meta-analyses to identify shared loci between AD and frailty. Our causality analysis provides no substantial evidence for a causal relationship between these two conditions. However, in gene-based association analysis, we found common genes attaining genome-wide cutoff for AD and frailty. Lastly, pathway analyses revealed a notable enrichment of lipoprotein particle plasma, amyloid fibril formation, protein kinase regulator, and tau protein binding for those genes common to AD and frailty.

## Materials and methods

**FIGURE 1:**
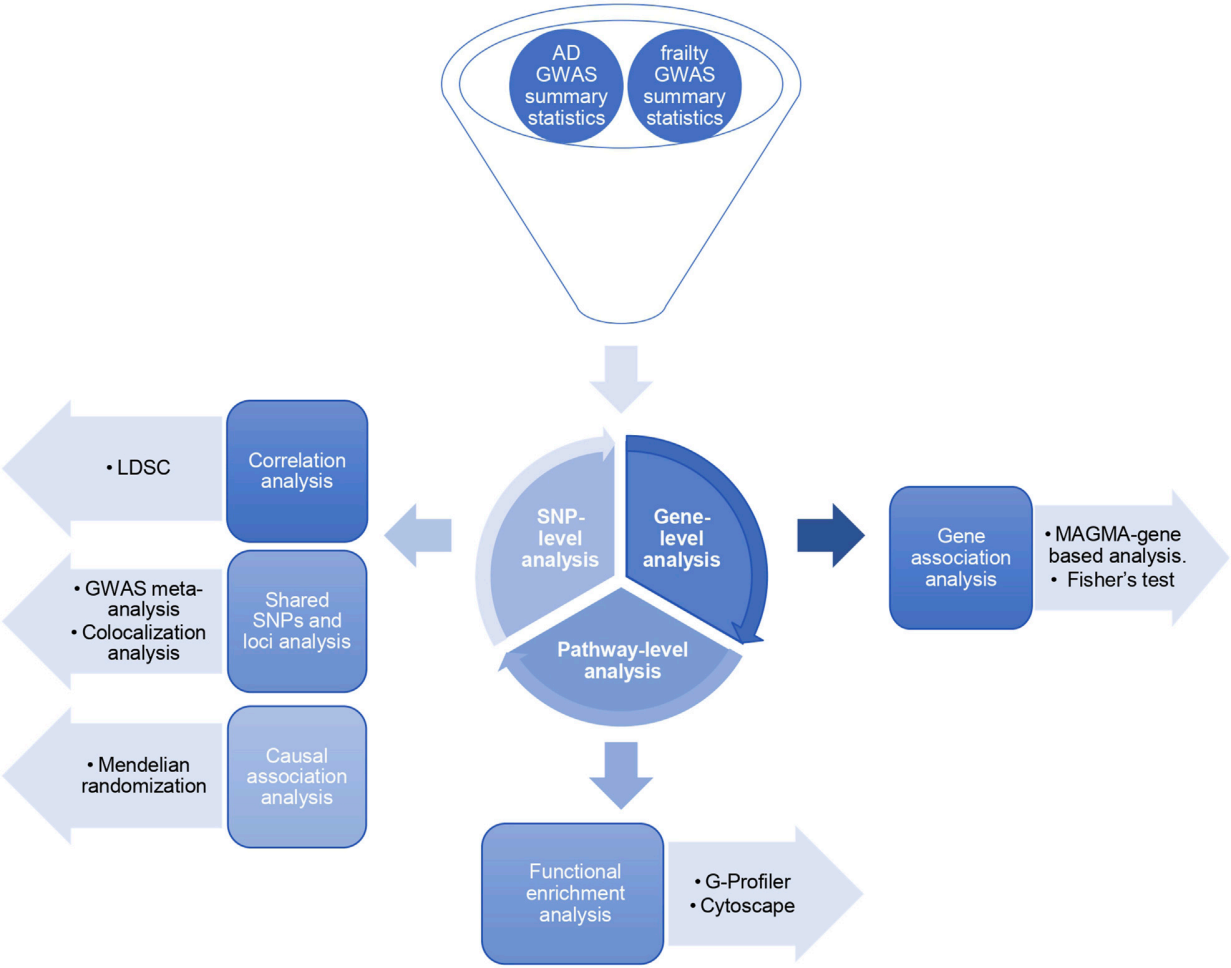
Study design. Three levels of SNP-based, gene-based, and pathway-based analyses exploring the shared genetic architecture between Alzheimer’s disease and frailty.

The general workflow of the study is summarized in Figure 1.

### GWAS summary statistics

We obtained GWAS summary statistics data for AD, which included only diagnosed AD individuals (n = 398,058) (Wightman et al., 2021) (GWAS data with AD cases only, excluding AD proxies from UK Biobank, was made available by the authors upon request). Separately, GWAS summary statistics on FI (n = 164,610) (Atkins et al., 2021) and FP (n = 386,565) (Ye et al., 2023) analyzing participants from the UK Biobank were obtained. All individuals were of European ancestry, and the summary statistics were built on the genome reference GRCh37. There was no sample overlap between AD and each of the frailty assessments (FI and FP) GWAS datasets (Supplementary Table S1).

### Linkage disequilibrium score regression (LDSC) between AD and frailty

To estimate the genetic correlation between AD and each of the frailty assessments, we employed the cross-trait linkage disequilibrium score regression (LDSC) method (Bulik-Sullivan et al., 2015). To ensure the robustness of our analysis, we utilized pre-estimated linkage disequilibrium (LD) scores provided by the developers of LDSC. These LD scores were derived from the 1,000 Genomes European reference population. We preprocessed the summary statistics using LDSC munge_sumstats.py and used the z-score as the signed summary statistic. To minimize the potential for bias arising from variations in LD structure, we conducted the genetic correlation calculations by incorporating HapMap3 SNPs in conjunction with LD reference panel SNPs. We excluded genomic regions covering the major histocompatibility complex (MHC, chr6: 25,119,106–33,854,733) and apolipoprotein E (*APOE*, chr19:44,000,000-47,000,000) from our analysis, considering their complex LD structure and known large genetic effect in AD, respectively.

### GWAS cross-trait meta-analysis

We conducted cross-trait meta-analyses of AD and each of the frailty assessments GWAS data to identify shared pleiotropic SNPs and loci between both traits. The fixed effect (FE) and the modified random effect (RE2) (Han and Eskin, 2011) models were implemented in our meta-analyses using the METASOFT program (http://genetics.cs.ucla.edu/meta/). The RE2 model operates under the assumption that SNP effects vary and that it computes *p* values via a likelihood ratio test, in contrast to the FE model, which assumes uniform effect sizes across studies. The FE derives the *p* values using the inverse variance weighted (IVW) effect size methodology, which may be inadequate in the presence of heterogeneity. Our primary objective with this approach was to unveil the SNPs that, while not initially meeting the genome-wide significance threshold (5 × 10^−8^ < *p_GWAS-SNP_* < 0.05), attained this level of significance following each meta-analysis (*p_meta-analysis_* < 5 × 10^−8^).

To further evaluate the shared SNPs between AD and each of the frailty assessments, we used the posterior probability (m-value) scores (Han and Eskin, 2011) from the meta-analysis results, which predict the effect size estimate in each of the studies under heterogeneity. An effect was predicted to exist if the posterior probability was greater than 0.9, no effect when the posterior probability was below 0.1, and an ambiguous effect when it was between 0.1 and 0.9 (Han and Eskin, 2011).

### Genomic loci definition and functional annotation

For AD and each of the frailty meta-analyses, independent SNPs and loci were identified based on the FUMA protocol, an online tool for functional mapping of genetic variants (http://fuma.ctglab.nl/) (Watanabe et al., 2017). As a result of our meta-analysis, SNPs that reached genome-wide significance (*p_meta-analysis_* < 5 × 10^−8^) with linkage disequilibrium (LD) *r* ^2^ < 0.6 with each other were recognized as independently significant SNPs. A subgroup of these independent SNPs with LD *r* ^2^ < 0.1 was then considered as the lead SNPs. The genomic locus boundaries were identified by detecting all the candidate SNPs that were in LD (*r* ^2^ ≥ 0.6) with the lead SNP. If the distance between two loci was smaller than 250 kb, they were merged. Some loci may have multiple LD-independent (r^2^ < 0.1) lead SNPs within the locus. All candidate SNPs located in these distinct locations were regarded as one independent genomic locus. The LD calculations were based on the 1000 Genomes Project (Clarke et al., 2017) reference panel for European ancestry.

We annotated the genes to the lead SNPs based on positional mapping using ANNOtate VARiation (ANNOVAR) (Wang et al., 2010) in FUMA. Additionally, these SNPs were annotated with Combined Annotation Dependent Depletion (CADD) scores (Rentzsch et al., 2019) to predict how certain the SNP effect is on protein structure or function and possible contribution to genetic disease. Similarly, we used RegulomeDB (RDB version 1.1) scores (Boyle et al., 2012) to predict the likelihood of regulatory functionality and chromatin states to predict transcription and regulatory effects from chromatin states at the SNP locus. RDB ranks the SNPs with a score from 1 (1a to 1f) to 6. The SNPs with strong evidence of being a regulatory variant are given a score of 1, and the ones with the least evidence are scored 6 (Boyle et al., 2012).

### Enrichment control and assessment of SNP novelty

SNPs within the MHC region (defined as chr6: 25,119,106 - 33,854,733) and *APOE* gene (chr19:44,000,000-47,000,000) were excluded from the analyses due to known association to AD (Lambert et al., 2010; Scheltens et al., 2016) or a very complex LD structure (de Bakker and Raychaudhuri, 2012). For the meta-analysis, we used a modified random effect (RE2) *p*-value and the GWAS *p*-value, i.e., *p_meta-analysis_* < 5 × 10^−8^ and 5 × 10^−8^ < *p_GWAS-SNP_* < 0.05 cutoff to identify the novel, previously unidentified shared SNPs between two traits in their original GWAS.

To assess their novelty, we examined our identified loci in previously reported GWAS associations in the National Human Genome Research Institute (NHGRI-EBI) GWAS Catalog (Sollis et al., 2023). We further identified if the gene was novel to any of our traits based on the traits reported in the GWAS Catalog.

### Colocalization analysis

To further investigate and discover genomic regions shared by each of the frailty assessment and AD, we conducted a colocalization study using the Pairwise GWAS approach (Pickrell et al., 2016) (GWAS-PW) (https://github.com/joepickrell/gwas-pw). The Bayesian pleiotropy association test, which reveals genomic regions that affect both traits, is the foundation of GWAS-PW. Additionally, we employed this technique to determine whether AD and frailty confidently share the same loci attaining genome-wide cutoff in our GWAS meta-analyses. The summary statistics of AD and each of the frailty assessments were combined using the GWAS-PW, and the posterior probability of association (PPA) of a pre-specified genomic region was calculated. GWAS-PW estimates four PPAs: i) the probability that the locus is associated with AD only (PPA1), ii) the probability that the locus is associated with frailty only (PPA2), iii) the probability that the locus is associated with both AD and frailty (PPA3) and iv) the probability that the locus is associated with both AD and frailty but through different causal variants (PPA4). The shared SNPs and regions (PPA3 and PPA4) were selected if their PPA >0.5 in the models.

### Causal relationship

We evaluated the potential causal relationship between AD and each of the frailty assessments using Mendelian randomization (MR) analyses. MR uses SNPs as instrument variables (IV) to assess the causality, and these IVs are defined by three assumptions (Davies et al., 2018). First, the selected IVs are significantly associated (*p*_GWAS_ < 5 × 10^−8^) with the exposure variable. Second, the IVs are independent between the exposure and the outcome. Third, the effect of IVs on the outcome must precede the exposure. We evaluated the bidirectional association between AD and each of the frailty assessments using the two-sample MR method (https://mrcieu.github.io/TwoSampleMR/articles/introduction.html). Initially, independent (*r* ^*2*^ < 0.001) genome-wide significant SNPs (*p*_GWAS_ < 5 × 10^−8^) associated with exposure (AD) were considered as instrumental variables (IVs) and assessed against outcome variables (FI and FP) and *vice versa*. We used the 5 MR methods (MR Egger, weighted median, inverse variance weighted (IVW), simple mode, and weighted mode) implemented in the 2SMR R package (Hemani et al., 2018).

### Gene-based association analysis

We carried out gene-based association analyses to find genome-wide significant genes shared between AD and each of the frailty assessments. By combining the effects of multiple SNPs, this methodology enhances SNP-based studies and increases the power for detecting genetic risk variations. It additionally addresses the problem of smaller effect sizes or correlations amongst SNPs. We used the multi-marker analysis of genomic annotation (MAGMA) (de Leeuw et al., 2015) software (https://ctg.cncr.nl/software/magma) to conduct the gene-based association analysis for overlapping SNPs between AD and each of the frailty assessments. To annotate the SNPs present within the gene region and to carry out precise gene-based testing, we used the gene boundary length as within ‘±0 kb outside the gene’ downstream and upstream.

Genome-wide significant genes for AD and FI were defined at a corrected *p*-value <2.79 × 10^−6^ (Bonferroni correction for qualified 17,919 genes, 0.05/17,919), based on the MAGMA results. With an adjusted *p*-value of 2.83 × 10^−6^ (Bonferroni correction for qualified 17,683 genes (0.05/17,683), we also discovered genome-wide significant genes for AD and FP. Additionally, we retrieved their overlapping genes at gene-level *p*-value <0.1 (*p*_gene_ < 0.1) to find the genes shared between AD and frailty. By implementing the Fisher’s combined *p*-value (FCP) analysis (Adewuyi et al., 2022) approach, we merged the FCP values for AD and frailty. We identified the common genes with genome-wide significance for AD and frailty at FCP <0.05.

### Pathway-based analysis

To explore the underlying biological mechanisms between AD and frailty, we performed overrepresentation analysis (ORA) using the g:GOSt tool from the g-profiler software (Raudvere et al., 2019), a web-based functional pathway analysis tool. To perform analysis using user specified genes, the g:GOSt tool includes databases Gene Ontology, Human Protein Atlas, Wikipathways, Human Phenotype Ontology, CORUM, Kyoto Encyclopedia of Genes (KEGG), and Reactome. We attempted to interpret gene functionality between AD and frailty based on these findings. We chose the genes that were the output of the gene-based association analysis as input for pathway analysis. We used the default functional parameter in the tool (Reimand et al., 2019) for our analysis. The range of acceptable functional category term sizes was 5–1,000. We used the default ‘g: SCS threshold’ (set counts and sizes) for multiple testing correction and reported the significantly enriched pathways at the multiple testing adjusted *p*-value <0.05. We used the enrichment map from Cytoscape (Shannon et al., 2003) to visualize the pathways with FDR <10^−3^ and auto-annotate cluster to annotate the pathways.

## Results

The workflow of our study is shown in Figure 1. We performed the analysis at three levels: SNP, gene, and pathway-based analysis (Adewuyi et al., 2022). First, in the SNP-based analysis, we computed the SNP-heritability and genetic correlation based on the LDSC method. The AD heritability was approximately 1.5% (*h* ^2^_SNP_ = 0.0155, SE = 0.0023), 12% for FI (*h* ^2^_SNP_ = 0.1169, SE = 0.0053), and 6% for FP (*h* ^2^_SNP_ = 0.0623, SE = 0.0027). There was no significant correlation between AD and FI (*r*_*g*_ = −0.045, SE = 0.05, *p* = 0.3) and between AD and FP (*r*_*g*_ = 0.01, SE = 0.04, *p* = 0.8). FI and FP have shown significant genetic correlation (*r*_*g*_ = 0.75, SE = 0.02, *p* = 8.7e-309). Next, we performed GWAS meta-analyses to identify shared SNPs and loci associated with AD and frailty. We also used the pairwise GWAS colocalization method across the predefined genomic loci to identify the loci with shared genetic influence between AD and frailty. We assessed for any probable causal relationship between AD and frailty using MR. Finally, we conducted gene and pathway-level-based analyses to find the genes reaching genome-wide significance and the biological pathways between AD and frailty.

### Cross-trait meta-analysis: shared and novel SNPs, loci, and genes between AD and frailty

We conducted cross-trait meta-analyses of AD and frailty utilizing GWAS summary statistics. We used a random effect meta-analysis to find SNPs that were not genome-wide significant in the individual AD or frailty GWAS (i.e., 5 × 10^−8^ < *p*_GWAS_ < 0.05) but reached statistical significance in our analysis (*p*_meta-analysis_ < 5 × 10^−8^). Our meta-analyses found shared SNPs, some of which were novel for AD or/and frailty (Table 1). In our cross-trait meta-analyses, the SNPs with genome-wide significance in these loci were not reported to be significant in their original GWAS.

**TABLE 1:** Genome-wide significant independent SNPs and loci for AD and frailty.

Locus	Lead SNP	Gene/cytoband	CHR:BP	A1:A2	RE2 p	AD m	Frailty m	AD p	Frailty p	AD z	Frailty z
SNPs and loci reaching genome-wide significance after meta-analysis of AD and frailty index (FI)											
1	rs35096827	CLRN1-AS1/3q25.1	3:150616348	T:C	2.09E-08	0.9	1	3.43E-02	8.58E-08	−2.11	−5.36
		RP11-166N6.2/3q25.1									
2	rs2071689	GRK4/4p16.3	4:3039311	T:C	2.52E-08	0.9	1	1.89E-02	1.57E-07	2.36	5.18
3	rs9471333	LRFN2/6p21.2	6:40362023	T:C	3.47E-08	0.9	1	4.08E-02	2.71E-07	−2.05	−5.18
4	rs11039165	MADD/11p11.2	11:47312689	A:G	3.77E-08	1	0.1	2.26E-07	9.60E-04	−5.17	−3.32
5	rs117500469	RP11-368P15.1/14q22.1	14:53428839	A:G	4.48E-10	1	1	1.20E-06	5.01E-05	4.85	4.06
		FERMT2/14q22.1									
6	rs1635298	CRHR1/17q21.31	17:43744344	A:T	1.84E-08	1	0.9	1.44E-05	1.60E-04	4.34	3.77
7	rs28483960	ZNF652/17q21.33	17:47432879	T:C	3.33E-09	0.9	0.7	2.90E-07	6.97E-05	5.15	4.00
8	rs11660554	LINC01929/18q21.2	18:52796510	A:G	2.39E-08	0.9	1	2.57E-03	6.90E-07	3.01	4.94
		RNA5SP459/18q21.2									
SNPs and loci reaching genome-wide significance after meta-analysis of AD and frailty phenotype (FP)											
1	rs6722241	AFF3/2q11.2	2:100803778	T:C	1.96E-08	0.9	1	4.64E-02	6.70E-08	−5.08	4.43
		LINC01104/2q11.2									
2	rs2515933	GRK4/4p16.3	4:3027897	C:G	3.38E-08	0.9	1	4.24E-02	9.70E-08	−2.05	−5.44
2	rs10012797	RGS12/4p16.3	4:3385176	A:G	2.54E-08	0.9	1	8.33E-03	1.60E-07	2.86	5.16
3	rs34647879	MAD1L1/7p22.3	7:1960646	A:G	1.40E-08	0.9	1	1.06E-02	6.80E-08	1.96	−5.49
4	rs11039307	C1QTNF4/11p11.2	11:47611152	T:C	1.69E-09	0.9	0.5	3.77E-07	8.10E-06	−2.03	−5.37
		FAM180B/11p11.2									
5	rs1785498	ZDHHC5/11q12.1	11:57448932	T:C	1.53E-08	0.9	1	4.03E-02	5.90E-08	1.99	5.42
6	rs34342224	CLEC16A/16p13.13	16:11225441	T:C	2.81E-08	0.9	1	4.26E-03	2.00E-07	2.03	5.29
7	rs35733741	FBXL19/16p11.2	16:30945887	A:T	1.22E-08	0.9	1	4.99E-02	5.10E-08	−2.64	−5.23
8	rs34979937	ZNF521/18q11.2	18:22690663	A:G	2.37E-08	0.9	1	4.25E-02	8.60E-08	−2.55	5.36

**FIGURE 2:**
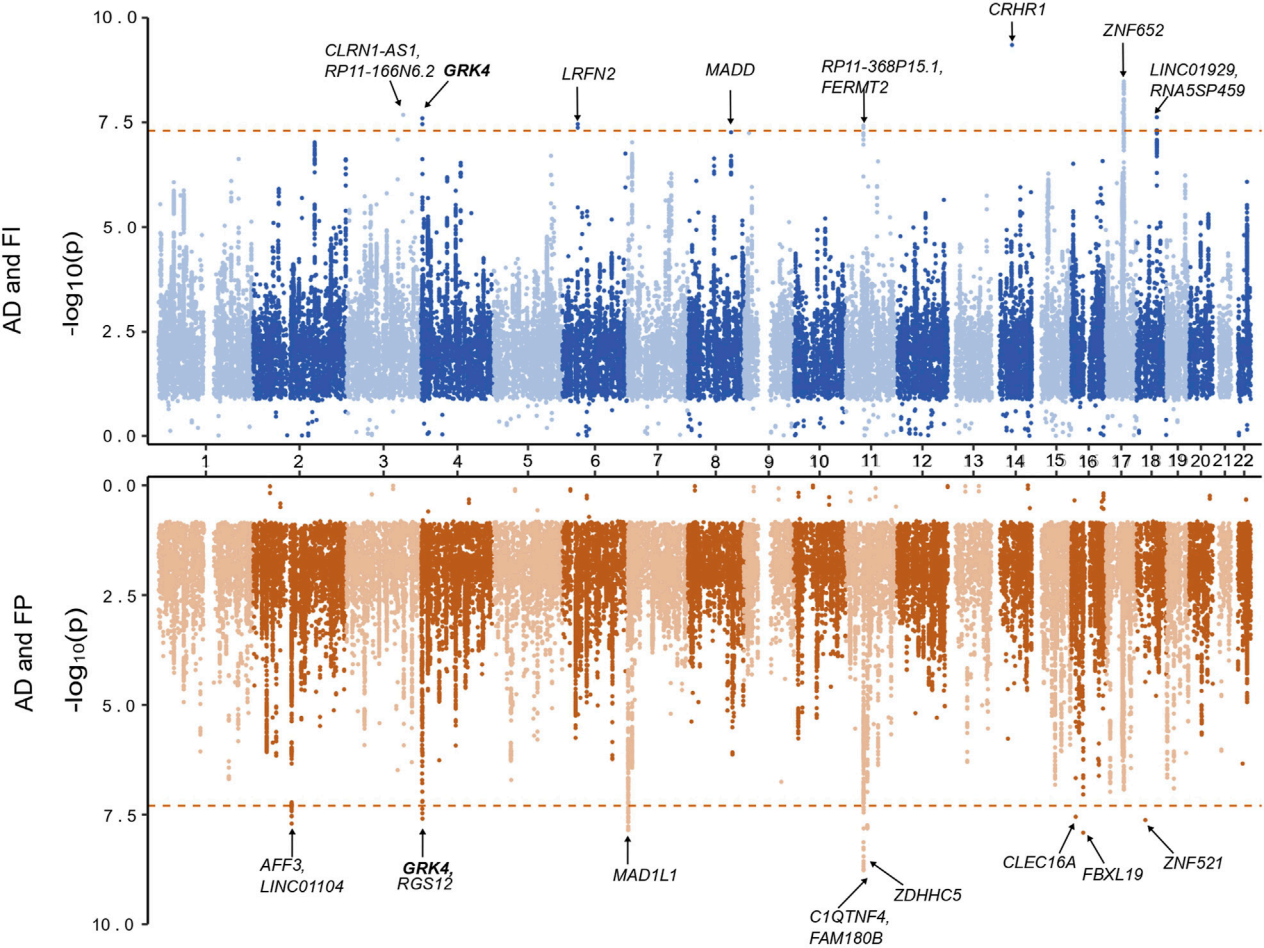
Miami plot based on the cross-trait meta-analyses of AD and frailty. The top plot is based on the meta-analysis of AD and the frailty index. The bottom plot is based on the meta-analysis of AD and frailty phenotype. The gene highlighted in bold is identified in both of the meta-analyses.

A total of 9,090,769 overlapping SNPs in the AD and FI GWAS were meta-analyzed in the combined 562,668 individuals. A total of 55 SNPs attained a genome-wide significance (Supplementary Table S2). To identify the independent and lead SNPs, we filtered them at LD r^2^ < 0.6 and subsequently at r^2^ < 0.1 in FUMA. Any overlapping SNPs within a 250 kb region were merged into a single locus, and the SNP with the least *p*-value was considered the lead SNP. We identified 8 genomic loci, each with a lead SNP. Of these 8 loci, 5 loci (3q25.1, 4p16.3, 6p21.2, 14q22.1, 18q21.2) were novel for both AD and FI, and 3 loci (11p11.2, 17q21.31, 17q21.33) were novel for FI (Table 1; Figure 2) and had pleiotropy association with AD. Based on the posterior probability (m-value), the effect exists for AD in all the SNPs, and, for FI, rs11039165 had no effect on FI, and rs28483960 had an ambiguous effect. These 8 lead SNPs were located in 11 genes (*CLRN1-AS1, CRHR1, FERMT2, GRK4, LINC01929, LRFN2, MADD, RP11-368P15.1, RP11-166N6.2, RNA5SP459, and ZNF652*), are either in their intronic (66.3%) or intergenic (33.7%) regions. All these genes are novel to FI. Five genes (*CLRN1-AS1, GRK4, LINC01929, RP11-368P15.1, RP11-166N6.2*) are novel to both AD and FI, and 3 (*FERMT2, LRFN2, ZNF652*) of them have been previously associated with AD or a family history of AD (Lambert et al., 2013; Marioni et al., 2018; Schwartzentruber et al., 2021; Wightman et al., 2021). The three RNA genes, *CLRN1-AS1, RP11-166N6.2,* and *RP11-368P15.1*, were not previously reported for any AD-related phenotype in the EBI GWAS Catalog.

Additionally, we identified 115 independent SNPs at 83 loci at the genome-wide suggestive association (*p*_meta-analysis_ < 1 × 10^−5^). Five of the 8 genome-wide significant loci (excluding loci: 3q25.1, 6p21.2, 14q22.1) were replicated at genome-wide suggestive association level for AD and FI (Supplementary Table S4). Also, we found SNPs and loci that were already known to be associated (*p*_GWAS_ < 5 × 10^−8^) with AD (AD lead SNPs) and, upon meta-analyses, were related to FI and *vice versa* (Supplementary Table S5).

Regarding the FP assessment, a total of 6,665,979 overlapping SNPs in the AD and FP GWAS were meta-analyzed in the combined 551,175 individuals. Meta-analysis of AD and FP identified 54 SNPs reaching a genome-wide significant association (Supplementary Table S3), and we identified 9 independent (*r* ^2^ < 0.1) genomic loci. Of these 9 loci, 8 were novel for both AD and FP, and one locus was novel for FP only (Table 1; Figure 2), which was also identified in a pleiotropy study between AD and gastroesophageal reflux disease (GERD). Eleven genes (*AFF3, C1QTNF4, CLEC16A, FAM180B, FBXL19, GRK4, LINC01104, MAD1L1, RGS12, ZDHHC5, and ZNF521*) were mapped to the 9 lead SNPs and their regions are either intronic (66.3%), intergenic (16.7%) or downstream (16.7%). All the genes are novel to FP, 8 of the genes are novel to both AD and FP (*MAD1L1* being the exception), and 3 genes (*AFF3, LINC01104, MAD1L1*) have been previously associated with the pleiotropy analysis of AD with educational attainment (EA) (Kulminski et al., 2022). Based on the posterior probability (m-value), the effect exists for both AD and FP in all the SNPs, and SNPS within the locus 11p11.2 (chr11:47611152—47232038) had an ambiguous effect on FP (Supplementary Table S3). The loci 4p16.3 and 11p11.2 and gene *GRK4* were shared with both pairs of AD and frailty.

We identified 120 independent SNPs at 79 loci at the genome-wide suggestive association (*p*_meta-analysis_ < 1 × 10^−5^). Seven of the 8 significant genome-wide loci (excluding locus 18q11.2) were replicated at genome-wide suggestive association levels for AD and FP (Supplementary Table S5). Additionally, we found SNPs and loci that were already known to be associated (P_GWAS_ < 5 × 10^−8^) with AD (AD lead SNPs) and, upon our meta-analyses, were related to FP too, and *vice versa* (see details in Supplementary Table S6).

### Colocalization analysis: shared loci across genomic regions

We assessed the genomic regions shared between AD and frailty using GWAS-PW (Supplementary Tables S7, S8). The findings of this research imply that AD and frailty share all the loci found in the meta-analyses with varying posterior probability (PPA4 > 0.2) (Table 2). The posterior probability result (PPA3 < 0.2) of causal variants suggests that those variants in the locus may be in strong LD, which restricts the GWAS-PW analysis ability to differentiate between model 3, where the locus is shared between both traits, from model 4, where the locus is shared between both traits, but through other causal variant (Pickrell et al., 2016). Additional shared genomic regions with PPA4 > 0.9 were identified in chromosomes 6, 10, and 11 for AD and FI, and PPA4 > 0.8 were identified in chromosomes 1, 3, 5, 6, 7, 11, 14, and 19 (Supplementary Table S7). For AD and FP, genomic regions in chromosomes 7, 10, 11, 14, and 19 had PPA4 > 0.9 and PPA4 > 0.8 in chromosomes 2, 5, 6, 10, 11, 15, 16, and 17 (Supplementary Table S8).

**TABLE 2:** Colocalization analysis on the meta-analysis significant loci between Alzheimer’s disease and frailty.

Locus	NSNP	CHR	Start	Stop	PPA_1	PPA_2	PPA_3	PPA_4
Pairwise analysis between Alzheimer’s disease and frailty index								
1	2680	chr3	150252004	151348193	8.60E-04	6.37E-01	2.79E-02	3.18E-01
2	2751	chr4	2844641	3844625	3.30E-07	7.51E-01	1.13E-02	2.38E-01
3	5,045	chr6	40345542	42038449	4.17E-03	3.43E-05	8.24E-04	9.95E-01
4	6,212	chr11	47008125	49865926	1.06E-01	1.33E-05	2.44E-02	8.69E-01
5	6,151	chr14	51493572	53473918	3.83E-02	1.62E-01	1.54E-02	7.02E-01
6	6,406	chr17	43056905	45874715	4.35E-03	4.30E-01	1.21E-02	5.20E-01
7	4076	chr17	45876022	47516523	1.66E-03	5.67E-01	1.70E-01	2.22E-01
8	8,459	chr18	51554436	55213381	9.31E-04	6.75E-01	4.40E-02	2.58E-01
Pairwise analysis between Alzheimer’s disease and frailty phenotype								
1	7,275	chr2	98995201	101822144	5.04E-08	4.69E-01	5.08E-02	4.81E-01
2	3,075	chr4	2845274	3845571	3.64E-12	2.94E-01	4.62E-01	2.45E-01
3	2557	chr7	1354471	2061783	8.80E-09	3.96E-01	1.14E-01	4.91E-01
4	7,878	chr11	47008125	49865926	4.67E-03	2.16E-07	7.53E-01	2.43E-01
5	12262	chr11	55082693	58455737	1.11E-06	6.00E-01	4.54E-02	3.54E-01
6	4118	chr16	10426040	11519750	1.08E-03	2.59E-01	4.88E-01	2.39E-01
7	2977	chr16	29038452	31379355	6.37E-05	1.65E-01	3.38E-02	8.01E-01
8	5,500	chr18	20649667	22994310	4.86E-05	5.98E-01	4.13E-02	3.59E-01

NSNP: number of Single nucleotide polymorphisms, CHR: chromosome, PPA_1: the probability that the locus is associated with AD, only, PPA_2: the probability that the locus is associated with frailty only, PPA_3: the probability that the locus is associated with both traits, PPA_4: the probability that the locus is associated with both traits but through different causal variants.

### Causal association analysis by MR

Since both AD and frailty are diagnosed in the later stage of aging population, it is cumbersome to determine the temporality between these disorders. Therefore, to evaluate the probable causal relationship between AD and frailty, we used the two-sample MR method. Regardless of the direction of the investigation, we employed the 5 MR methods [MR Egger, weighted median, inverse variance weighted (IVW), simple mode, and weighted mode] implemented in the 2SMR R package. Although, no statistically significant evidence was detected for a causal association between AD and frailty (AD as outcome and frailty as exposure, and *vice versa*) within all the tests (Table 3), the effect of the variants is significant on these combined traits.

**TABLE 3:** Summary of Mendelian randomization analysis results between Alzheimer’s disease and frailty.

Exposure	Outcome	Method	# SNPs	b	se	p
Mendelian randomization between Alzheimer’s disease and frailty index						
FI	AD	MR Egger	15	1.73	1.08	0.13
		Weighted median	15	−0.11	0.20	0.59
		Inverse variance weighted	15	−0.13	0.15	0.37
		Simple mode	15	0.23	0.36	0.53
		Weighted mode	15	0.15	0.39	0.70
AD	FI	MR Egger	22	−0.03	0.03	0.33
		Weighted median	22	−0.01	0.01	0.35
		Inverse variance weighted	22	−0.01	0.01	0.32
		Simple mode	22	0.00	0.02	0.89
		Weighted mode	22	−0.01	0.02	0.42
Mendelian randomization between Alzheimer’s disease and frailty phenotype						
FP	AD	MR Egger	36	0.99	0.80	0.22
		Weighted median	36	−0.15	0.22	0.51
		Inverse variance weighted	36	−0.22	0.20	0.26
		Simple mode	36	−0.18	0.45	0.68
		Weighted mode	36	−0.22	0.40	0.59
AD	FP	MR Egger	27	−0.01	0.37	0.97
		Weighted median	27	−0.03	0.02	0.12
		Inverse variance weighted	27	−0.03	0.02	0.09
		Simple mode	27	−0.03	0.31	0.93
		Weighted mode	27	−0.03	0.03	0.38

AD: Alzheimer’s disease, FI: frailty index, FP: frailty phenotype, b: beta, se: standard error, p: *p*-value.

### Gene association analysis identified AD-related genes

Using the overlapping SNPs between AD and FI, we performed gene-based analysis using MAGMA. We found a total of 17,919 protein-coding genes shared between AD and FI. By applying Bonferroni correction *p*-value cutoff (0.05/17919, 2.79 × 10^−6^), we identified 37 protein coding genome-wide significant genes for AD (Supplementary Table S9) and 43 for FI (Supplementary Table S10). A total of 17 genes (*p*_gene_ < 2.79 × 10^−6^) shared between AD and FI were found by using the FCP technique (Supplementary Table S13), 16 of which were significant (*p*_gene_ < 2.79 × 10^−6^). All these genes are novel for FI, and 3 of those genes are novel for both AD and FI (Supplementary Table S13). Eleven genes (*ANCA7, ARHGAP45, C17orf107, CD33, CLU, CRHR1, KANSL1, LAMB2, MAPT, PICALM, SPPL2c*) have been previously related to AD in the EBI GWAS Catalog. We found 2 overlapping loci (4p16.3 and 17q21.31) between gene analysis and SNP meta-analysis but no overlapping gene.

We found 17,683 protein-coding genes shared between AD and FP. After applying the Bonferroni correction (0.05/17683, *p*-value = 2.83 × 10^−6^), we identified 40 genome-wide significant genes for AD (Supplementary Table S11) and 86 for FP (Supplementary Table S12). A total of 41 shared genes were found using the FCP technique (Supplementary Table S14). All these genes were significant at the *p*_gene_ < 2.83 × 10^−6^ level. All these genes were novel for FP, and 22 were novel for both AD and FP (Supplementary Table S14). Eighteen genes (*ADAM10, ATXN2L, BCL1A, CD33, CELF1, CRHR1, EPHA1, FNBP4, KANSL1, LAMB2, MAD1L1, MAPT, MS4A3, NUP160, PICALM, PSMC3, SPI1, and SPPL2C*) were previously related to AD in the EBI GWAS Catalog. There were 3 overlapping loci (4p16.3, 7p22.3, and 11p11.2) and genes (*C1QTNF4, MAD1L1,* and *RGS12*) between the gene analysis and SNP meta-analysis. CD33, *CRHR1, KANSL1*, *LAMB2,* and *MAPT* were the overlapping genes between FI and FP, with AD based on the SNP and gene-level analysis.

### Pathway-based analysis

**FIGURE 3:**
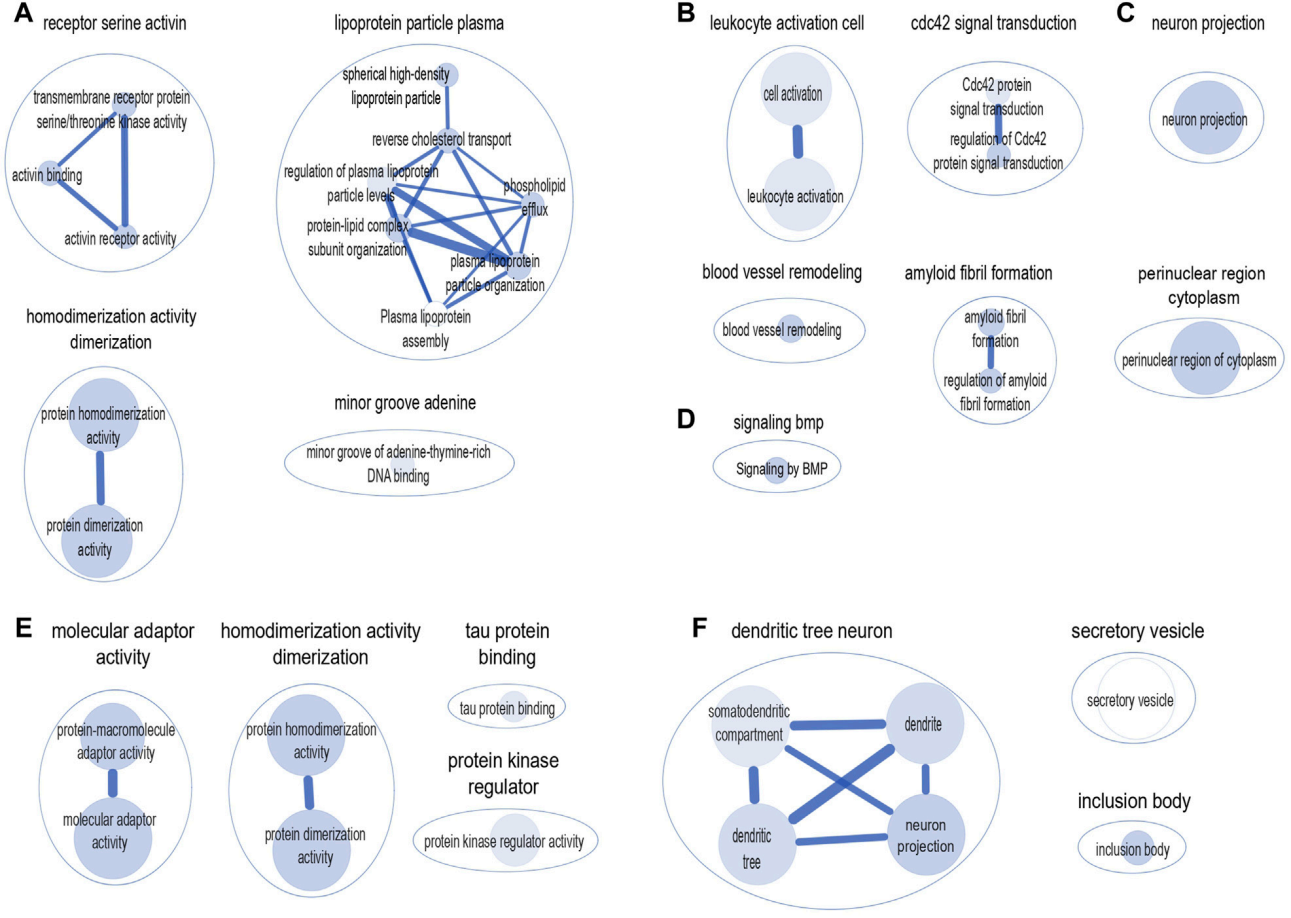
Clusters of biological pathways enriched with the genes shared between AD and frailty. Pathways enriched with the genes shared between AD and frailty index: **(A)** Gene Ontology: Molecular Function. **(B)** Gene Ontology: Biological Pathway. **(C)** Gene Ontology: Cellular Components. **(D)** Reactome: Biological Pathway. Pathways enriched with the genes shared between AD and frailty phenotype: **(E)** Gene Ontology: Molecular Function. **(F)** Gene Ontology: Cellular Components. The size of the inner circle represents the number of genes within the pathway. The bigger the circle, the higher the number of genes associated with that pathway. The color darkness of the inner circle denotes the strength of the *p*-value. The higher the *p*-value, the lighter the color of the circle.

We used the g: Profiler, a web-based platform to perform pathway-based enrichment analysis to functionally interpret the genes that overlap between AD and frailty. We examined the genes associated with AD and frailty (*p*_gene_ < 0.1, FCP <0.05) (Supplementary Table S15, S16). We found multiple enriched biological pathways, suggesting a shared role in the biological mechanisms between AD and FI. This analysis identified 50 significantly enriched biological pathways or processes, mostly related to receptor activity, amyloid fibril formation, lipoprotein particle, and leukocyte activation. These include serine/threonine kinase activity (*p*_adjusted_ = 1.18 × 10^−4^), protein homodimerization activity (*p*_adjusted_ = 6.12 × 10^−4^), plasma lipoprotein particle organization (*p*_adjusted_ = 8.81 × 10^−5^), Cdc42 protein signal transduction (*p*_adjusted_ = 5.46 × 10^−4^), amyloid fibril formation (*p*_adjusted_ = 2.86 × 10^−8^), among others (Supplementary Table S17). Cytoscape identified six major clusters: ‘homodimerization activity dimerization,’ ‘receptor serine activin,’ ‘lipoprotein particle plasma,’ ‘leukocyte activation cell,’ ‘cdc42 signal transduction’, and ‘amyloid fibril formation’ (Figure 3).

Similarly, between AD and FP, 18 biological pathways were identified, which include ‘protein-macromolecule adaptor activity’ (*p*_adjusted_ = 1.25 × 10^−4^), ‘protein homodimerization activity’ (*p*_adjusted_ = 2.11 × 10^−4^), and ‘tau protein binding’ (*p*_adjusted_ = 4.51 × 10^−4^) (Supplementary Table S18). Cytoscape shows four major clusters: ‘molecular adaptor activity’, ‘homodimerization activity dimerization’, ‘protein kinase regulator’, and ‘tau protein binding’ (Figure 3). Of note, the ‘homodimerization pathway’ was shared between the two frailty assessments with AD.

## Discussion

Epidemiological studies have reported comorbidity of aging disorders like AD with frailty, but no previous research has focused on the shared genetic architecture between AD and frailty. Similarly, in a cross-sectional study in mice models, increased frailty has been seen in AD (Kapphan et al., 2023). In addition, observational studies in humans have suggested a shared relationship between AD biomarkers and frailty (Wallace et al., 2018). In this study, we comprehensively assessed the genetic overlap, causal relationship, shared genes, and biological pathway between AD and frailty using GWAS summary statistics. Similar to the previous observational studies, our findings suggested a shared genetic architecture between AD and frailty.

In our analyses, LDSC did not provide a significant genetic correlation between AD and frailty, which may be due to the mixed effect of the influence of SNPs on each phenotype. The genetic correlation between FI and FP was 0.75. We did not find studies measuring the phenotypic correlation between FI and FP in the UK biobank population. To the best of our knowledge, longitudinal studies based on the European ancestry population showed a modest kappa agreement ranging from 0.38 to 0.45 (Thompson et al., 2018; Kim et al., 2022). The kappa score >0.8 suggests a strong agreement. The genetic correlation method, like LDSC, failed to capture the mixture of effect directions across the shared variants, i.e., nullifying the negative effect of the variant on one trait and the positive effect of the variant on another trait (Frei et al., 2019). Since the LDSC approach cannot reveal the completely shared variants associated with AD and frailty, we followed a multi-statistical approach to identify shared variants and genes at the SNP and gene levels and performed the pathway analysis to explore the shared biological mechanism.

The cross-trait meta-analyses identified 8 loci shared between AD and FI; among them, 3 (*FERMT2, LRFN2, ZNF652*) were previously associated with AD. The loci 11p11.2 (*MADD*) and 17q21.31 (*CRHR1*) were previously reported in the pleiotropy relationship between AD and EA (Kulminski et al., 2022). The loci 17q21.33 (*ZNF652*) was previously reported in the pleiotropic relationship between AD and GERD (Adewuyi et al., 2022). The cross-trait analysis of AD and FP identified 9 loci, 8 of which were novel for both AD and FP, and 1 locus was novel for FP only. All the genes were novel for FP, and 3 genes (*AFF3, LINC01104,* and *MAD1L1*) were in a pleiotropy relationship between AD and EA. The lead SNP rs1635298 (mapped to 17q21.31, *CRHR1*) has a RegulomeDB score of 1f, which suggests regulatory functionality. SNP rs1785498 (11q12.1, *ZDHHC5*) has a CADD score of 20, suggesting its deleteriousness. The colocalization analysis showed a moderate to high (20%-99%) probability of association signals at the loci identified from the meta-analyses and high association signals in the regions of chromosomes 1, 2, 3, 5, 6, 7, 11, 14, 15, 16, and 19 between AD and frailty. However, these association signals were not sufficiently strong to provide any bidirectional causal relationship between AD and frailty. The gene analyses by Fisher’s test identified several loci (4p16.3, 7p22.3, 11p11.2, and 17q21.31) and genes (*RGS12, MAD1L1,* and *C1QTNF4*) that were shared with the meta-analyses results.

Of note, there were several genes (*AFF3, CRHR1, FERMT2, GRK4, LINC01104, LRFN2, MADD, MAD1L1,* and *ZNF652*) identified in the meta-analyses were associated with AD or had a pleiotropic relationship with AD and its risk factors such as obesity-related traits (Comuzzie et al., 2012), cognitive performance (Lee et al., 2018), EA (Okbay et al., 2022), hypertension (Hoffmann et al., 2017), immune diseases (Johansson et al., 2019), neuropsychiatric disorders (Yao et al., 2021), and BMI (Tachmazidou et al., 2017). We discussed some of them below.

*AFF3* is a putative transcription activator involved in oncogenesis and lymphoid development. It has been associated with immune diseases like rheumatoid arthritis and type 1 diabetes, plays role in controlling immune responses and protects against infections (Tsukumo et al., 2022). *CRHR1*, which showed regulatory functionality in our analyses, encodes a Gprotein coupled receptor for CRH (corticotropin-releasing factor) and UCN (urocortin). It is related to GPCR downstream signaling and activation of cAMP-dependent Protein kinase A (PKA) pathways. Activation of *CRHR1* by CRH has been shown to increase the release of APP in rat cerebellar neurons, in human neuroblastoma IMR32 cell line, and in mouse hippocampal HT22 cells (Lezoualc’h et al., 2000). The reduction in CRH levels is associated with morphological abnormalities in brain areas affected by AD (Bissette et al., 1985; De Souza et al., 1986). Downregulation of the cAMP-response element-binding protein (CREB) in AD brains has been linked to cognitive and memory impairments (Liang et al., 2007). *FERMT2* (encoding fermitin family member 2) is a scaffolding protein mediated by *TLN1* and/or *TLN2* that enhances integrin activation and plays a role in the TGFB1 and integrin signaling pathways. *FERMT2*, implicated in tau metabolism (Shulman et al., 2014), has also been associated with AD risk factors such as systolic and diastolic blood pressures (Hoffmann et al., 2017). The 4p16.3 (*GRK4*) locus was shared between AD and frailty. GRK4 (G Protein-Coupled Receptor Kinase 4), a paralog for GRK5, has been associated with AD pathology (Guimaraes et al., 2021) and is a potential drug target (Zhang et al., 2022). *LINC01104,* an RNA gene, has a pleiotropic relationship between AD and EA (Kulminski et al., 2022). *LRFN2*, encoding Leucine Rich Repeat and Fibronectin Type III Domain Containing 2, is a protein-coding gene that promotes neurite outgrowth in hippocampal neurons and processes the frequency of synaptic transmission from a neuron to a target cell across a synapse. A proteomic study of synaptic markers has shown a strong association of *LRFN2* with cognitive decline in an AD population (Bereczki et al., 2018). *LRFN2* is also associated with obesity-related traits (Comuzzie et al., 2012), cognitive performance (Lee et al., 2018), EA (Okbay et al., 2022), psychiatric conditions such as insomnia (Watanabe et al., 2022) and schizophrenia (Trubetskoy et al., 2022), and multiple cancers (lung cancer, gastric cancer, and squamous cell carcinoma) (Jin et al., 2012). *MADD* (MAP Kinase Activating Death Domain protein) belongs to the DENN protein family. It regulates the Rab family of small GTPases. *MADD* acts as a guanine nucleotide exchange factor (GEF) for Rab3, which is present on synaptic vesicles and regulates neurotransmitter release. *MADD* has been associated with several diseases, including Deeah syndrome, which affects developmental, intellectual growth, and neurodevelopmental disorders with dysmorphic facies, impaired speech, and hypotonia. *MADD* is associated with glycemic traits (blood insulin and glucose measurement) (Masotti et al., 2019), which impacts insulin resistance in depressed people, a common risk factor for AD and frailty (Fernandes et al., 2022). It is also involved in several pathways, including the TNF signaling and TNFR1 pathway. The TNFR1 and MADD proteins interact and mediate downstream protein signaling pathways that result in neuronal cell death and AD, possibly being drug targets for AD (Hassan et al., 2021). *MAD1L1* is a part of the spindle-assembly checkpoint that delays the beginning of anaphase until all chromosomes are correctly positioned at the metaphase plate (Jin et al., 1999; Nakano et al., 2010; Ji et al., 2018). Neurodevelopmental processes in mice and human organoids have shown a role for *MAD1L1* in the impairment of neuronal migration and neurite outgrowth (Goo et al., 2023). Gene *ZNF652* is predicted to enable DNA-binding transcription factor activity, RNA polymerase II-specific, and RNA polymerase II cis-regulatory region sequence-specific DNA binding activity. It has been identified as a risk gene for hypertension (Hoffmann et al., 2017), one of the known AD risk factors. *ZNF652* is associated with allergic diseases such as asthma, eczema, and allergic rhinitis (Johansson et al., 2019), and it was reported to be associated in a pleiotropic relationship between AD and asthma (Enduru et al., 2024).

The gene analyses cumulatively identified 77 genes for AD and 129 genes for frailty (includes both FI and FP). Overall, 58 genes were identified between AD and frailty at FCP cutoff and they help in the understanding of their shared biological mechanisms. Since frailty is classified based on multi-comorbidities, using the genes associated with AD and frailty can help to identify novel drug targets for multiple diseases and offer more treatment options for these comorbidities.

Our pathway enrichment analysis of the genes shared between AD and frailty revealed several pathways, including ‘Activin binding,’ ‘amyloid fibril formation,’ and ‘lipoprotein and dendrite tree neurons.’ Activins are members of the transforming growth factor β (TGFβ) family and play a pivotal role in signal transduction across the central nervous system (CNS). They serve as multifunctional regulatory proteins in many tissues and organs (Link et al., 2016). Activins can also activate pathways like ‘mitogen-activated protein kinase (MAPK) signaling’ (Moustakas and Heldin, 2005), which are involved in the pathophysiology and pathogenesis of AD (Zhu et al., 2002). Activin type II receptor, part of the TGFβ family has been related to loss of muscle in aging population and heart failure severity, the risk factors associated to frailty (Roh et al., 2019). ‘Amyloid fibrils’ are self-assembled fibrous protein structures of β-rich forms (Aβ_1-40_), linked to many currently incurable disorders, including AD and Parkinson’s disease (Ow and Dunstan, 2014). Studies have shown that neuronal disruption with the Aβ fibril formation results in symptoms similar to AD (Paola et al., 2000). Aβ has been a prominent protein in the study of amyloid fibril formation (AFF). Many therapeutics have focused on developing inhibitors capable of preventing AFF (Hardy and Selkoe, 2002; Liu et al., 2022) by using antibodies against AFF (Hardy and Selkoe, 2002; Mangialasche et al., 2010) and decreasing the production of Aβ, all of which could possibly halt the progression of AD (Hardy and Selkoe, 2002; Lichtenthaler, 2011). Varying plasma lipoprotein cholesterol levels of ApoA and ApoC have been suggested to alter regional brain volumes related to AD. Higher cholesterol/ApoA ratios were linked to lower cortical grey matter volume and higher ventricular volume. In contrast, higher ApoA and ApoJ/ApoA ratios were linked to higher cortical grey matter volume (and for ApoA-II, higher hippocampal volume) and lower ventricular volume (Pedrini et al., 2022). The shared ‘dendrite tree neuron pathway’ between AD and FI provides evidence for the association of excessive neuron loss and regression in dendrites in both AD and frailty (Coleman and Flood, 1987; Cochran et al., 2014).

In our study, some pathophysiology mechanisms have been identified and several biological pathways, both in each condition in isolation and shared between AD and frailty are reported, such as the inflammation pathway, which reported kynurenine pathway in association with AD (Fernandes et al., 2023) and frailty (Kim et al., 2020). Several observational studies reported the relationship between brain pathologies and frailty. Previous study showed rapid progression of frailty in the presence of pathologies related to macroinfarcts, AD, Lewy body and nigral neuronal loss (Buchman et al., 2013). Other similar studies have shown the accumulation of Aβ from cerebrospinal fluid to be associated to worsening frailty (Yoon et al., 2018; Maltais et al., 2019). In this line, our analysis provided evidence of genetic overlap between AD and frailty, similarly to observational studies that have suggested a consistent comorbid association between AD and frailty. Studies suggested that those with higher amount of frailty were more likely to have more AD pathology, which later are expressed as dementia (Wallace et al., 2019; Ward et al., 2022).

A key aspect of this study is the use of various complementary statistical genetic techniques, which allowed for a deep analysis of the genetic relationships between AD and frailty. We used only clinically diagnosed cases of AD to reduce the possibility of false-positive and false-negative findings. In addition, we used well-powered GWAS, which is considerably less affected by the small sample size frequently observed in traditional observational studies. Our results highlight the shared genomic loci between AD and frailty with minimal genetic correlation and no causality. Our study comes with some limitations. First, diagnosing frailty is challenging. The FI is based on health deficits accumulated during the life course, and the specific health deficits may vary from person to person. A minimum number of accumulated health deficits must be considered to adequately calculate the FI. The FP is based on the presence of three out of five physical components (weakness, slow walking speed, inadequate physical activity, exhaustion, and unexpected weight loss), and there may be misdiagnosis based on the participant’s responses to health questionnaires. Our analyses were restricted to only individuals of European ancestry; thus, our results may not be representative of other populations. Further genetic studies are needed to validate and refine our findings. For example, additional GWAS and genome sequencing data from cohorts ascertaining these phenotypes and the related large datasets from general cohorts such as UK Biobank and All of Us could provide further evidence to support of our findings. In addition, other types of omics data such as epigenetic data can provide additional biological evidence regarding genetic relationships. Finally, animal models and cell lines can provide additional tools to validate the potential function and phenotypic outcomes by functional genetic studies, including mutation knock in or out in mice.

To conclude, our study explores the shared genetic relationship between the two aging-related conditions, AD and frailty. Our study utilized the 54 frailty items which focused on various mental health conditions, cardiovascular diseases, immune-mediated diseases, pain, and physical frailty to mention a few (5 FP, 49 FI items) in the aging population. It provides some novel insights into the shared genetic architecture from SNP and gene-level to biological pathways that are associated with AD and frailty. Our findings based on SNP analyses show minimal genetic correlation but reveal significant shared loci and genes between AD and frailty, from gene-based analyses. These shared loci present a significant shared genetic association between AD and frailty with a varying posterior probability, with several loci (4p16.3, 7p22.3, 11p11.2, and 17q21.31) and genes (*RGS12, MAD1L1,* and *C1QTNF4*) being shared in the SNP meta-analyses and gene analyses. Finally, we identified several biological pathways common to AD and frailty. This is the first genetic study exploring the genetic and biological relationship between AD and frailty using extensive yet complementary statistical approaches. Overall, our findings provide evidence of genetic relationship between the commonly co-occurring conditions of AD and frailty.

## Web resources

The software’s used in this study are freely available here: Genetic correlation analysis: https://github.com/bulik/ldsc.

Cross-trait analysis: http://genetics.cs.ucla.edu/meta colocalization analysis: https://github.com/joepickrell/gwas-pw mendelian randomization: https://mrcieu.github.io/TwoSampleMR/

MAGMA: https://ctg.cncr.nl/software/magma gprofiler: https://biit.cs.ut.ee/gprofiler/gost.

Cytoscape: https://cytoscape.org/

## Data Availability

Publicly available datasets were analyzed in this study. This data can be found here: The GWAS summary statistics for Alzheimer’s disease and frailty phenotype are available through the GWAS catalog (https://www.ebi.ac.uk/gwas/) under accession no. GCST013196 and GCST90020053. The GWAS summary statistics for frailty index is freely available in [https://figshare.com/articles/dataset/Genome-Wide_Association_Study_of_the_Frailty_Index_-_Atkins_et_al_2019/9204998].
